# Effects of interference with GATA-3 expression by target-specific DNAzyme treatment on disease progression in a subacute oxazolone-induced mouse model of atopic dermatitis

**DOI:** 10.1186/2045-7022-5-S1-O21

**Published:** 2015-03-11

**Authors:** Rouba Ibrahim, Ulrich Purath, Agnieszka Turowska, Ursula Homburg, Frank Runkel, Thomas Schmidts, Dorota Dobler, Harald Renz, Holger Garn, Anke Mueller

**Affiliations:** 1 Philipps University of Marburg, Inst. of Laboratory Medicine and Pathobiochemistry, Marburg, Germany; 2 sterna biologicals, ZTI, Marburg, Germany; 3 University of Applied Sciences, IBPT, Giessen, Germany

## Background

DNAzymes represent a particular class of antisense molecules combining the specificity of antisense molecules with an inherent catalytic cleavage activity, which makes them an attractive tool for highly specific interference with target RNA molecules. In general, they are single-stranded DNA molecules with sequence-specific RNA-binding domains flanking a central catalytic domain. We developed and patented a DNAzyme - named hgd40 – that targets the mRNA for GATA-3, the central transcription factor in T helper cell type 2 (Th2) differentiation and activation. For penetration enhancement and DNAzyme protection a specific water/oil/water emulsion for topical dermal application was developed and patented. Targeting GATA-3 might be a key for therapeutic intervention in predominantly Th2-driven diseases like atopic dermatitis.

## Method

The therapeutic effects of hgd40 were analyzed in an oxazolone-induced dermatitis model modified to establish elongated skin swelling reactions, thereby enabling the analysis of treatment effects on T cell-mediated pathomechanisms.

## Results

Treatment with topically applied hgd40 water/oil/water emulsion significantly and dose-dependently reduced oxazolone-induced skinfold thickness and suppressed infiltration of CD4+ T cells into the skin. Molecular analysis revealed reduced GATA-3 mRNA levels early during disease progression.

## Conclusion

In summary, targeting GATA-3 by DNAzyme treatment may represent a new and promising therapeutic agent for the topical treatment of allergic skin diseases.

